# A novel monoclonal antibody against the von Willebrand Factor A2 domain reduces its cleavage by ADAMTS13

**DOI:** 10.1186/s13045-017-0407-1

**Published:** 2017-02-06

**Authors:** Lulu Zhang, Jian Su, Fei Shen, Zhenni Ma, Yiming Zhao, Lijun Xia, Changgeng Ruan

**Affiliations:** 1 Jiangsu Institute of Hematology, Key Laboratory of Thrombosis and Hemostasis of Ministry of Health, The First Affiliated Hospital of Soochow University, Suzhou, 215006 China; 2 Collaborative Innovation Center of Hematology, Soochow University, Suzhou, 215006 China

**Keywords:** von Willebrand factor, Monoclonal antibody, ADAMTS13

## Abstract

We developed a novel murine monoclonal antibody (mAb) against the C-terminal α-helix of the human von Willebrand factor A2, designated SZ-179. We showed that SZ-179 inhibited the interactions between VWF and ADAMTS13 and prevented the degradation of high molecular weight VWF multimers. Importantly, SZ-179 reduced the proteolysis of VWF-R1597W mutant by rADAMTS13 dose-dependently under native conditions. Our findings reveal a potential therapeutic target for bleeding disorders.

**Electronic supplementary material:**

The online version of this article (doi:10.1186/s13045-017-0407-1) contains supplementary material, which is available to authorized users.

## Findings

ADAMTS13 (a disintegrin and metalloproteinase with a thrombospondin type 1 motif, member 13) regulates the multimeric size of von Willebrand factor (VWF) by cleaving the Tyr1605-Met1606 bond in the VWF A2 domain (VWFA2) [1]. This remarkable cleavage specificity depends largely on the binding of the noncatalytic ADAMTS13 spacer domain to the C-terminal α-helix of VWFA2 [2]. A 73 amino acid residue from D1596 to R1668 in VWF A2 domain, designated VWF73, serves as a minimal substrate for ADAMTS13 [3]. In concert, deletion of the VWFA2 C-terminal α-helix (E1660-R1668) from this minimal substrate leads to nearly complete loss of cleavage by ADAMTS13, indicating that this structure is essential to the binding and cleavage of VWF by ADAMTS13 [4, 5].

**Fig. 1:**
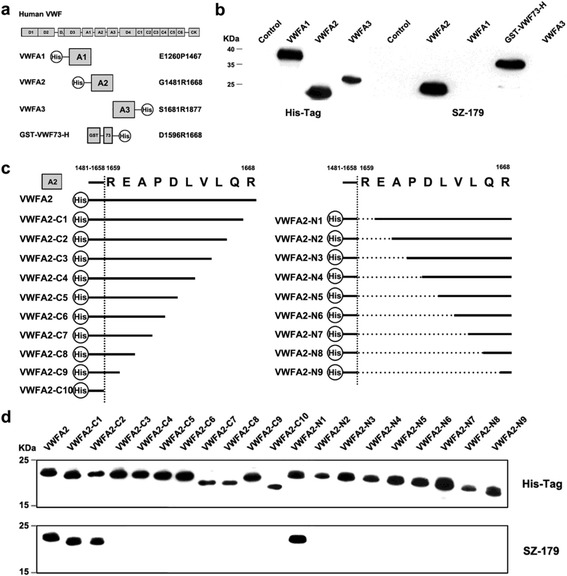
Epitope mapping of SZ-179. **a** Schematic analysis of the human VWF. Domains are indicated. **b** VWFA1, VWFA2, GST-VWF73-H, and VWFA3 were separated by reduced 15% SDS-PAGE and detected with a mouse anti-His antibody (*left*) or SZ-179 (*right*). **c** Schematic analysis of 20 different VWFA2 mutants with His tags. **d** VWFA2 and its deletion mutants were separated by 15% reducing SDS-PAGE and detected by Western blotting with anti-His (*top*) or SZ-179 (*bottom*)

We utilized standard hybridoma technology to develop monoclonal antibodies (mAbs) that detects the A2 domain of VWF (Additional file 1). One mAb (9G11), designated SZ-179, was identified as an immunoglobulin G1 (IgG1) subtype. SZ-179 interacts with both the synthetic R1659-R1668 peptide (VWFα5) and native VWF with high affinity (50 ng/ml), as determined by enzyme-linked immunosorbent assay (ELISA) (Additional file 2: Figure S1). To identify the epitope of SZ-179, we evaluated the binding of this mAb to distinct VWF fragments, including VWFA1 (H-E1260P1467), VWFA2 (H-G1481R1668), VWFA3 (S1681R1877-H), and GST-VWF73-H (GST-D1596R1668-H) (Fig. 1a). As expected, SZ-179 bound to VWFA2 and GST-VWF73-H rather than VWFA1 or VWFA3 (Fig. 1b). These results suggest that SZ-179 mAb specifically targets the A2 domain of VWF. To further pinpoint the epitope recognized by SZ-179, we generated a series of VWFA2 deletion mutants (Fig. 1c). The interactions of SZ-179 with VWFA2 and its deleted versions were determined by Western blotting. The results indicated that SZ-179 specifically bound to only VWFA2 (H-G1481R1668), VWFA2-C1 (H-G1481Q1667), -C2 (H-G1481L1666) and -N1(H-G1481P1658-E1660R1668) (Fig. 1d). These findings suggest that the epitope of SZ-179 is located within the distal portion of the VWFA2 domain between amino acid residues E1660-L1666.

**Fig. 2:**
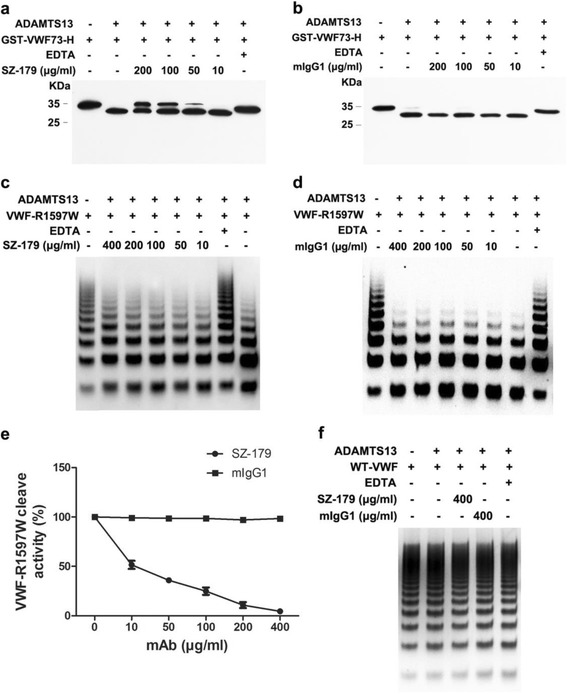
SZ-179 inhibits rADAMTS13-mediated cleavage of the minimal substrate GSH-VWF73-H and VWF-R1597W under native conditions. **a**, **b** GST-VWF73-H (2.8 μg) was pre-incubated with SZ-179 or isotype IgG1 for 2 h at 37°C and then incubated with 50 nM rADAMTS13 for 1 h. The cleavage products were analyzed by 15% reducing SDS-PAGE and Western blotting with an HRP-conjugated mouse anti-GST antibody. **c**, **d** VWF-R1597W (150 nM) was incubated with SZ-179 or isotype control murine IgG1 at 4 °C for 30 min, followed by 3 h with 50 nM rADAMTS13 at 4 °C. The proteolytic products were separated by electrophoresis in a 1.3% agarose gel and detected by anti-VWF. **e** Dose–response curve for inhibition of rADAMTS13-mediated cleavage of VWF-R1597W. **f** Wild-type VWF (150 nM) was treated with rADAMTS13 at 4 °C for 3 h as a control. Results represented as mean ± SD of four independent experiments

Next, we determined if SZ-179 affects rADAMTS13-mediated cleavage of the minimal substrate. We found that SZ-179, but not the isotype control murine IgG1, inhibited GST-VWF73-H cleavage by rADAMTS13 dose-dependently (Fig. 2a, b), with a half maximal inhibitory concentration (IC50) of 221.8 μg/ml (Additional file 3: Figure S2). In this light, SZ-179 abrogates cleavage of a minimal VWF substrate by rADAMTS13.

Moreover, we found that pre-incubation of plasma with SZ-179 rather than with the isotype control resulted in a dose-dependent decrease in the proteolysis of high molecular weight (HMW) VWF multimers under static/denaturing conditions, with an IC50 of 0.66 μg/ml (Additional file 3: Figure S2). These findings suggested that SZ-179 can bind to native VWF and provided further evidence that SZ-179 may attenuate the susceptibility of VWF to proteolytic cleavage by ADAMTS13 under physiological conditions.

We next determined whether SZ-179 could inhibit rADAMTS13-mediated proteolysis of the VWF-R1597W mutant, which can be cleaved by ADAMTS13 under static conditions and in the absence of denaturants including urea and guanidine [6, 7]. The R1597W mutation is commonly associated with von Willebrand disease (VWD) type 2A and located within VWFA2, close to the ADAMTS13 cleavage site. We found that the proteolysis of HMW VWF-R1597W multimers by rADAMTS13 was dramatically reduced by SZ-179 rather than by IgG1 isotype control in a concentration-dependent manner under native conditions (Fig. 2c, d). The IC50 of SZ-179 for this reaction was 13.54 μg/ml (Fig. 2e). Nevertheless, wild-type VWF treated with rADAMTS13 remained intact, as expected in the absence of chemical denaturation or fluid shear stress (Fig. 2f). These findings suggest that SZ-179 inhibits the rADAMTS13-mediated proteolysis of VWF-R1597W multimers under native conditions.

Mechanistically, SZ-179 may interact with E1660-L1666 residues in the VWF, blocking the binding of the spacer domain of ADAMTS13 to the substrate, thereby inhibiting proteolysis of VWF by ADAMTS13. Several recent reports support this possibility. For example, human neutrophil peptides inhibit ADAMTS13-dependent VWF proteolysis by binding to the central A2 domain of VWF to block interactions between ADAMTS13 and VWF [8]. Antibody mAb508 is specific to the D4 domain of VWF, and has been observed to interfere with ADAMTS13-mediated degradation of VWF in a vortex-based degradation assay [9]. mAb508 is bound to VWF with moderate affinity, and its binding to VWF partially inhibits the interaction between VWF and ADAMTS13. We discovered that SZ-179 has high affinity (50 ng/ml) with native VWF and prevents excessive degradation of HMW-VWF-multimers under denaturing conditions dose-dependently. SZ-179 may provide a promising therapeutic approach for a subset of VWD patients.

## Additional files

Additional file 1: Supplemental data. Detailed methods and materials are shown. (DOC 115 kb)
Additional file 2: Figure S1. Characterization of mAb SZ-179. (A) Quantification of ELISA analyses detecting SZ-179 binding to IgG1, IgG2a, IgG2a, IgG3, or IgM. (B) Quantification of ELISA analyses for SZ-179 or murine IgG1 binding to VWFα5. Dose–response curves are shown. (C) Quantification of ELISA analyses for SZ-179 or murine IgG1 binding to plasma-derived VWF. Dose–response curves are shown. Data are mean ± SD of four independent experiments. (DOCX 974 kb)
Additional file 3: Figure S2. SZ-179 inhibits cleavage of VWF by ADAMTS13 in plasma under denaturing conditions. (A, B) Pooled normal human plasma was pre-incubated with SZ-179 or isotype control IgG1 for 2 h at 37°C, and then incubated with 1.5M urea for 16 h. The proteolytic products were separated by electrophoresis in a 1.3% agarose gel and detected by anti-VWF. (C) Dose–response curve for inhibition of plasma ADAMTS13-mediated cleavage of plasma-VWF. (D) Dose–response curve for inhibition of rADAMTS13-mediated GST-VWF73-H cleavage. Results represented as mean ± SD of four independent experiments. (DOCX 1519 kb)

## Abbreviations

ADAMTS13: A disintegrin and metalloproteinase with a thrombospondin type 1 motif, member 13
ELISA: Enzyme-linked immunosorbent assay
HMW: High molecular weight
IC50: Half maximal inhibitory concentration
IgG1: Immunoglobulin G1
mAb: Murine monoclonal antibody
VWD: von Willebrand disease
VWF: von Willebrand factor
