# Supplementary material for: A novel monoclonal antibody against the von Willebrand Factor A2 domain reduces its cleavage by ADAMTS13
Source: J Hematol Oncol. 2017 Feb 6;10:42. doi: 10.1186/s13045-017-0407-1 (PMC5292787; doi:10.1186/s13045-017-0407-1)
Supplement: Additional file 1: — Supplemental data. Detailed methods and materials are shown. (DOC 115 kb) [file 13045_2017_407_MOESM1_ESM.doc]

**Additional file 1**

**Methods**

**Production and characterization of mAbs against VWF domain A2**

Balb/c mice were immunized by four injections of a synthetic peptide encompassing VWF residues R1659–R1668 (VWFα5, 100 μg/injection, Genscript, Nanjing, China) at 21-day intervals. The Freund’s complete adjuvant (Sigma-Aldrich) was used for the initial injection, with Freund’s incomplete adjuvant (Sigma-Aldrich) used for the second and third injections, and deionized water for the final injection. The first three immunizations were administered subcutaneously while the final one was given intravenously. Three days after the final immunization, mouse spleens were removed and splenocytes were isolated for cell fusion with SP2/0-Ag14 myeloma cell line (kindly provided by Blood Transfusion Hybridoma Laboratory Center, Paris, France). Hybridomas were selected in HAT culture medium (Thermo Fisher Scientific). Cultures producing antibodies specific for both VWFα5 and plasma-derived human VWF (purified from commercial VWF/FⅧ concentrate by gel filtration on a Sepharose 4B-CL column (TransGen Biotech) were identified and cloned by two rounds of culture under limiting-dilution conditions . Immunoglobulin G（IgG）was purified from ascites using a protein G-Sepharose 4B column.

The Ig isotype of SZ-179 was determined by an enzyme-linked immunosorbent assay (ELISA) according to manufacturer’s recommendation (Sino Biological). IgG1, IgG2a, IgG3 or IgM (1 μg/ml) were coated onto microtiter plates. Specific interactions were detected with horseradish peroxidase (HRP)-conjugated rabbit anti-mouse IgG (1:5000).

The binding activity of SZ-179 to VWFα5 and VWF was measured by ELISA. Ninety-six-well microtiter plates were coated overnight at 4 °C with 100 μl of VWFα5 (1 μg/ml) or plasma-derived human VWF, respectively. After three washes, the wells were blocked with 2% (w/v) bovine serum albumin (BSA) (AMRESCO) dissolved in phosphate-buffered saline (PBS) for 2 h. The wells were then incubated in purified SZ-179 or its isotype control murine IgG1 (Sigma-Aldrich) (10-1000 ng/ml) for another 2 h, followed by incubation with HRP-conjugated goat anti-mouse IgG (1:5000) (Santa Cruz Biotechnology). Binding was detected with tetramethyl benzidine (TMB)-hydrogen peroxide (H_2_O_2_) substrate (Thermo Fisher Scientific). The reaction was stopped by addition of 2 M H_2_SO_4_, and absorbance in each well was measured at a wavelength of 450 nm.

**Preparation of recombinant human VWF fragments**

Recombinant VWF fragments including VWFA1 (H-E1260P1467), VWFA2 (H-G1481R1668), VWFA3 (S1681R1877-H), GST-VWF73-H (GST-D1596R1668-H), VWFA2-C1 (H-G1481Q1667), VWFA2-C2 (H-G1481L1666), VWFA2-C3 (H-G1481V1665), VWFA2-C4 (H-G1481L1664), VWFA2-C5 (H-G1481D1663), VWFA2-C6 (H-G1481P1662), VWFA2-C7 (H-G1481A1661), VWFA2-C8 (H-G1481E1660), VWFA2-C9 (H-G1481R1659), VWFA2-C10 (H-G1481P1658), VWFA2-N1 (H-G1481P1658-E1660R1668), VWF-N2 (H-G1481P1658-A1661R1668), VWF-N3 (H-G1481P1658-P1662R1668), VWF-N4 (H-G1481P1658-D1663R1668), VWF-N5 (H-G1481P1658-L1664R1668), VWF-N6 (H-G1481P1658-V1665R1668), VWF-N7 (H-G1481P1658-L1666R1668), VWF-N8 (H-G1481P1658-Q1667R1668), and VWF-N9 (H-G1481P1658-R1668) were expressed in *Escherichia coli*. Plasmids encoding different VWF fragments were generated from pSVHVWF1, which harbors a full-length cDNA insert of human VWF (kindly provided by JE Sadler, Washington University School of Medicine, St Louis, USA) [2].

Plasmids encoding VWFA2 and a series of deletion mutants were constructed using a forward primer that contains a BamHI site and different reverse primers that contain HindIII sites (Table 1). After sequence analysis, amplified DNA fragments were digested with BamHI and HindIII (New England Biolabs) and ligated into the corresponding sites of the expression vector pQE30 (Qiagen), which contains a 6×His tag. *Escherichia coli* M15 was transformed with the recombinant expression vectors and expression induced with 0.5 mmol/L isopropyl β-D-1-thiogalactopyranoside (IPTG). Recombinant proteins were separated by 15% reducing sodium dodecylsulfate-polyacrylamide gel electrophoresis (SDS-PAGE). Gels were stained with Coomassie brilliant blue fast staining solution (TIANGEN Biotech) or transferred to a nitrocellulose membrane (PALL). The membranes were subsequently blocked with 5 % non-fat milk and incubated with a mouse anti-His antibody (1:1000, Immunotech) at 4 °C overnight. The membranes were then washed and incubated with HRP-conjugated goat anti-mouse IgG (1:5000) for 1.5 h at room temperature before development. Proteins were detected by enhanced chemiluminescence substrate according to the manufacturer’s instructions.

**Epitope mapping of SZ-179**

VWFA1, A3 and GST-VWF73-H, tagged with 6×His (NOVAGEN), were prepared as described previously . Recombinant VWFA2 (and deleted versions) were prepared as described above. Proteins were separated by 15% reducing SDS-PAGE, transferred to a nitrocellulose membrane, and immunoblotted with either SZ-179 (100 ng/ml) or mouse anti-His antibody (1:1000) (as positive control) for 2 h. SZ-179 or mouse anti-His were detected by HRP-conjugated goat anti-mouse IgG (1:5000).

**Proteolysis of GST-VWF73-H by recombinant ADAMTS13 (rADAMTS13)**

Plasmid containing cDNA encoding full-length human ADAMTS13 was generously provided by Dr. Jingfei Dong (University of Washington School of Medicine, Seattle, WA, USA). Recombinant ADAMTS13 with C-terminal His-tag was expressed in a stably transfected HeLa cell line as described previously .

Recombinant ADAMTS13 was activated by incubation with 5 mM calcium chloride dehydrate (CaCl_2_) (Sigma-Aldrich) in 20 mM Tris-HCl (pH 8.0) for 1 h. Purified VWF73 (2.8 μg) was pre-incubated with SZ-179 (0-200 μg/ml) or isotype control IgG1 (0-200 μg/ml) in a total volume of 40 μl in the presence of 20 mM Tris-HCl (pH 8.0 ) for 2h at 37°C, followed by treatment with activated 50 nM rADAMTS13 in 20 mM Tris-HCl (pH 8.0) for another 1 h. The reaction was quenched by adding 20 mM ethylene diamine tetra acetic acid (EDTA) (Sigma-Aldrich). The cleavage products were separated by 15% reduced SDS-PAGE and analyzed by western blotting with an HRP-conjugated mouse anti-GST antibody (1:1000) (Immunotech). Densitometry analysis was performed using Image J software (version 1.47). For each lane, the extent of cleavage was calculated by measuring the intensity of the cleavage product relative to the total intensity of distinguishable bands. Data were analyzed using Graph Pad Prism 5.0 software, and the half maximal inhibitory concentration (IC50) values were calculated using sigmoidal dose response curve fitting. Results were mean values of four independent experiments.

**Cleavage of native VWF by ADAMTS13 under denaturing conditions**

Pooled human plasma from 20 healthy individuals was pre-incubated with SZ-179 (0-10 μg/ml) or isotype control IgG1 (0-10 μg/ml) for 2 h at 37°C, followed by incubation with 1.5 M Urea in 20 mM Tris-HCl (pH 8.0) for 16 h. The reaction was stopped by adding 20 mM EDTA. The proteolytic products/VWF multimers were separated by 1.3% agarose gel electrophoresis. Gels were fixed on Gelbond (LONZA) and VWF multimeric sizes were detected with HRP-conjugated polyclonal rabbit-anti-human VWF antibody (1:3000) (Dako). Densitometry analysis was performed with Image J software. For each lane, the signal of the lowest band was calculated relative to density of the lowest band observed in the absence of SZ-179, and presented as a percentage. IC50 calculation was performed as described above.

**Cleavage of VWF-R1597W by rADAMTS13 under native conditions**

The plasmid PSVHvWF1 was used to generate the R1597W mutant by PCR-based single-nucleotide mutagenesis. The plasmids were transiently transfected into human embryonic kidney (HEK293) cell line using serum-free OPTIMEMI (Invitrogen), and recombinant proteins were expressed as described previously [6]. Recombinant VWF-R1597W (150 nM) was incubated with SZ-179 (0-400 μg/ml) or isotype control IgG1 (0-400 μg/ml), respectively, at 4°C for 30 min prior to incubation with 50 nM rADAMTS13 at 4°C for 3h. Recombinant wild-type VWF (150 nM) treated with 50 nM rADAMTS13 at 4°C for 3 h served as the control. The reaction was terminated by addition of 20 mM EDTA. Cleavage reactions were analyzed as described above.

**Reference**s

1. Kohler G, Milstein C. Continuous cultures of fused cells secreting antibody of predefined specificity. Nature. 1975;256:495-7.

2. Jorieux S, Tuley EA, Gaucher C, Mazurier C, Sadler JE. The mutation Arg (53)----Trp causes von Willebrand disease Normandy by abolishing binding to factor VIII. Studies with recombinant von Willebrand factor. Blood. 1992;79:563-7.

3. Kokame K, Matsumoto M, Fujimura Y, Miyata T. VWF73, a region from D1596 to R1668 of von Willebrand factor, provides a minimal substrate for ADAMTS-13. Blood. 2004;103:607-12.

4. Zhu H, Wang Y, Jiang M, Ji S, Bai X, Ruan C. Generation and characterization of a recombinant single chain Fv antibody to von Willebrand factor A1 domain from phage display library. Thromb Res. 2005;116:385-91.

5. Zhao Y, Dong N, Shen F, Xie L, He Y, Liu F, et al. Two novel monoclonal antibodies to VWFA3 inhibit VWF-collagen and VWF-platelet interactions. J Thromb Haemost. 2007;5:1963-70.

6. Zhang J, Ma Z, Dong N, Liu F, Su J, Zhao Y, et al. A conformation-sensitive monoclonal antibody against the A2 domain of von Willebrand factor reduces its proteolysis by ADAMTS13. PLoS One. 2011;6:e22157.

**Table 1.** Primers were applied to construction of plasmid VWFA2 and its mutants

| **Designation** | **Primer sequence** |
| --- | --- |
| **VWFA2** | AG**GGATCC**GGGCTCTTGGGGGTTTCGAC |
|  | AG**AAGCTT**TTACCTCTGCAGCACCAGGTCAG |
| **VWFA2-C1** | AG**GGATCC**GGGCTCTTGGGGGTTTCGAC |
|  | AG**AAGCTT**TTACTGCAGCACCAGGTCAGGAG |
| **VWFA2-C2** | AG**GGATCC**GGGCTCTTGGGGGTTTCGAC |
|  | AG**AAGCTT**TTACAGCACCAGGTCAGGAGCCT |
| **VWFA2-C3** | AG**GGATCC**GGGCTCTTGGGGGTTTCGAC |
|  | AG**AAGCTT**TTACACCAGGTCAGGAGCCTCTC |
| **VWFA2-C4** | AG**GGATCC**GGGCTCTTGGGGGTTTCGAC |
|  | AG**AAGCTT**TTACAGGTCAGGAGCCTCTCGG |
| **VWFA2-C5** | AG**GGATCC**GGGCTCTTGGGGGTTTCGAC |
|  | AG**AAGCTT**TTAGTCAGGAGCCTCTCGGGGGAG |
| **VWFA2-C6** | AG**GGATCC**GGGCTCTTGGGGGTTTCGAC |
|  | AG**AAGCTT**TTAAGGAGCCTCTCGGGGGAGC |
| **VWFA2-C7** | AG**GGATCC**GGGCTCTTGGGGGTTTCGAC |
|  | AG**AAGCTT**TTAAGCCTCTCGGGGGAGCGTC |
| **VWFA2-C8** | AG**GGATCC**GGGCTCTTGGGGGTTTCGAC |
|  | AG**AAGCTT**TTACTCTCGGGGGAGCGTCTCAAA |
| **VWFA2-C9** | AG**GGATCC**GGGCTCTTGGGGGTTTCGAC |
|  | AG**AAGCTT**TTATCGGGGGAGCGTCTCAAAGTC |
| **VWFA2-C10** | AG**GGATCC**GGGCTCTTGGGGGTTTCGAC |
|  | AG**AAGCTT**TTAGGGGAGCGTCTCAAAGTCCTG |
| **VWFA2-N1** | AG**GGATCC**GGGCTCTTGGGGGTTTCGAC |
|  | AG**AAGCTT**TTACCTCTGCAGCACCAGGTCAGGAGCCTCGGGGAGCGTCTCAAAGTCCTGGATGAG |
| **VWFA2-N2** | AG**GGATCC**GGGCTCTTGGGGGTTTCGAC |
|  | AG**AAGCTT**TTACCTCTGCAGCACCAGGTCAGGAGCGGGGAGCGTCTCAAAGTCCTGGATGAG |
| **VWFA2-N3** | AG**GGATCC**GGGCTCTTGGGGGTTTCGAC |
|  | AG**AAGCTT**TTACCTCTGCAGCACCAGGTCAGGGGGGAGCGTCTCAAAGTCCTGGATGAG |
| **VWFA2-N4** | AG**GGATCC**GGGCTCTTGGGGGTTTCGAC |
|  | AG**AAGCTT**TTACCTCTGCAGCACCAGGTCGGGGAGCGTCTCAAAGTCCTGGATGAG |
| **VWFA2-N5** | AG**GGATCC**GGGCTCTTGGGGGTTTCGAC |
|  | AG**AAGCTT**TTACCTCTGCAGCACCAGGGGGAGCGTCTCAAAGTCCTGGATGAG |
| **VWFA2-N6** | AG**GGATCC**GGGCTCTTGGGGGTTTCGAC |
|  | AG**AAGCTT**TTACCTCTGCAGCACGGGGAGCGTCTCAAAGTCCTGGATGAG |
| **VWFA2-N7** | AG**GGATCC**GGGCTCTTGGGGGTTTCGAC |
|  | AG**AAGCTT**TTACCTCTGCAGGGGGAGCGTCTCAAAGTCCTGGATGAG |
| **VWFA2-N8** | AG**GGATCC**GGGCTCTTGGGGGTTTCGAC |
|  | AG**AAGCTT**TTACCTCTGGGGGAGCGTCTCAAAGTCCTGGATGAG |
| **VWFA2-N9** | AG**GGATCC**GGGCTCTTGGGGGTTTCGAC |
|  | AG**AAGCTT**TTACCTGGGGAGCGTCTCAAAGTCCTGGATGAG |

Table 1 should appear at line 18, page 3, before the paragraph entitled *Epitope mapping of SZ-179.*
