# Supplementary figures and images for: A novel monoclonal antibody against the von Willebrand Factor A2 domain reduces its cleavage by ADAMTS13
Source: J Hematol Oncol. 2017 Feb 6;10:42. doi: 10.1186/s13045-017-0407-1 (PMC5292787; doi:10.1186/s13045-017-0407-1)

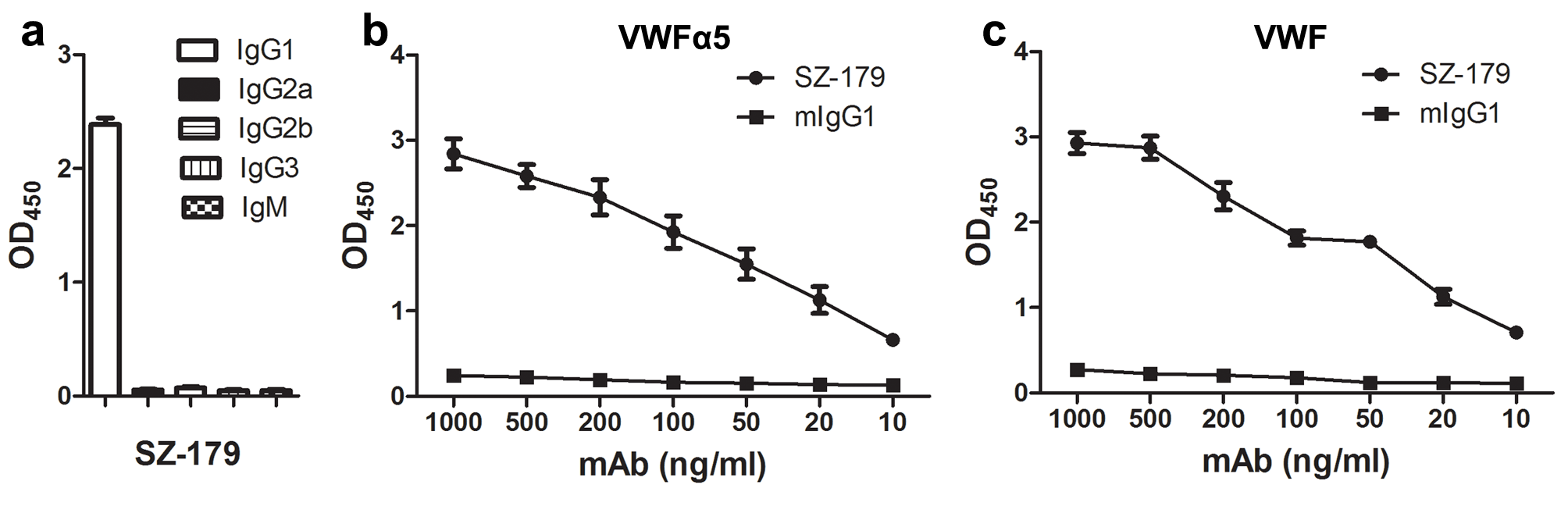


Additional file 2: Figure S1

Supplement: Additional file 2: Figure S1. — Characterization of mAb SZ-179. (A) Quantification of ELISA analyses detecting SZ-179 binding to IgG1, IgG2a, IgG2a, IgG3, or IgM. (B) Quantification of ELISA analyses for SZ-179 or murine IgG1 binding to VWFα5. Dose–response curves are shown. (C) Quantification of ELISA analyses for SZ-179 or murine IgG1 binding to plasma-derived VWF. Dose–response curves are shown. Data are mean ± SD of four independent experiments. (DOCX 974 kb) [file 13045_2017_407_MOESM2_ESM.docx]

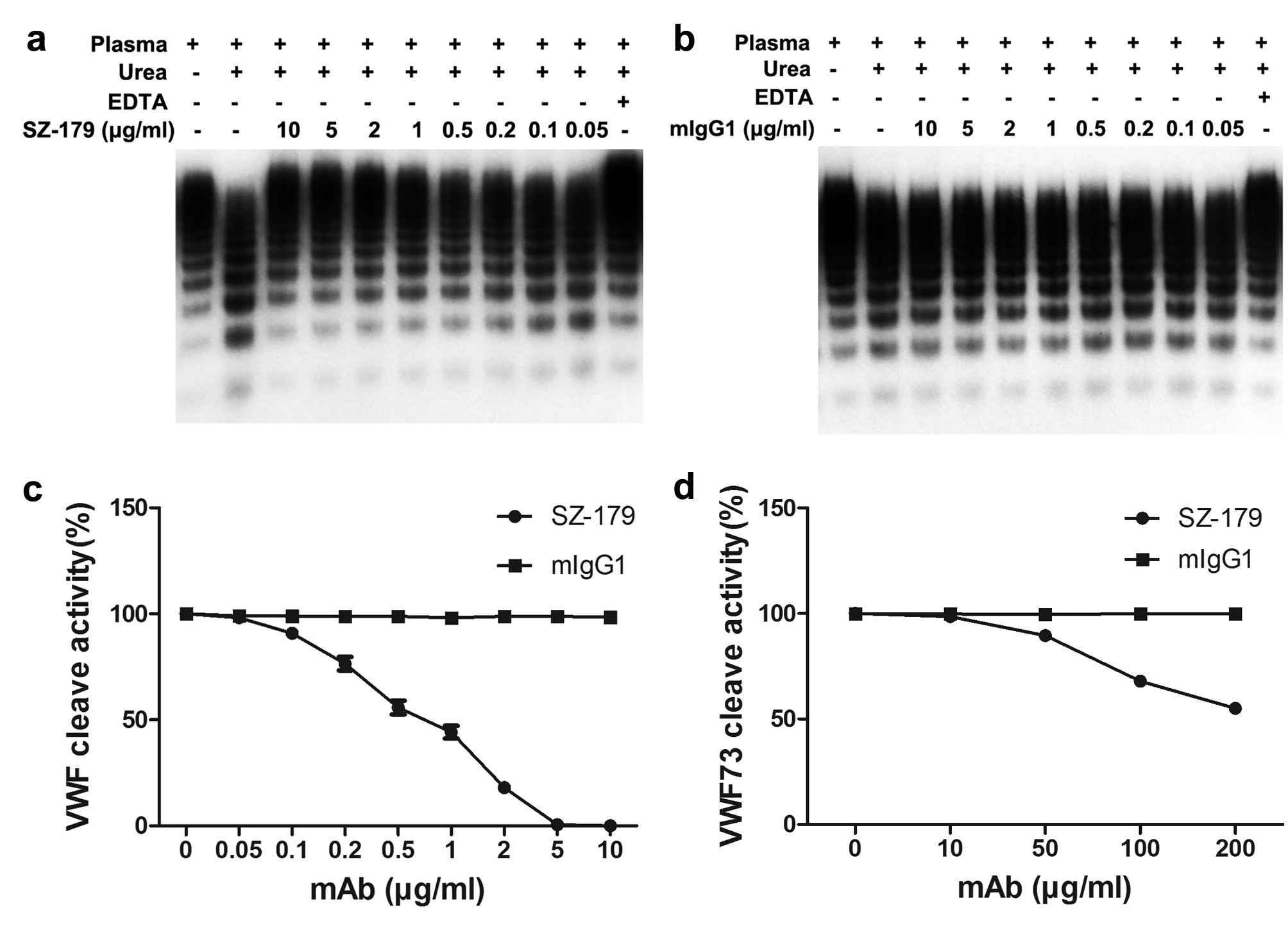


Additional file 3: Figure S2

Supplement: Additional file 3: Figure S2. — SZ-179 inhibits cleavage of VWF by ADAMTS13 in plasma under denaturing conditions. (A, B) Pooled normal human plasma was pre-incubated with SZ-179 or isotype control IgG1 for 2 h at 37°C, and then incubated with 1.5M urea for 16 h. The proteolytic products were separated by electrophoresis in a 1.3% agarose gel and detected by anti-VWF. (C) Dose–response curve for inhibition of plasma ADAMTS13-mediated cleavage of plasma-VWF. (D) Dose–response curve for inhibition of rADAMTS13-mediated GST-VWF73-H cleavage. Results represented as mean ± SD of four independent experiments. (DOCX 1519 kb) [file 13045_2017_407_MOESM3_ESM.docx]
